# Annotations

**Published:** 1910-11-05

**Authors:** 


### ANNOTATIONS.

### An Echo of the War.

The St. Andrew's Ambulance Association has decided: "That a Provisional Order be applied for by the Association to enable it to transfer the Red Cross Fund held by it to the Scottish Branch, British Red Cross Society, with the exception of a sum of £3,500, part of the said fund, which is to be retained by the Association to enable it to carry out obligations already undertaken, including those under the sanction of the War Office." Sir George T. Beatson has explained the object of this decision by reminding the recent annual meeting of the Association that the Scottish National Red Cross Hospital was raised at the time of the South African War mainly by its own efforts, and that £48,000 was subscribed through a general committee representative of Scotland. When the time came to divide the residue, £9,000 fell to the St. Andrew's Ambulance Association, which was responsible for its employment in the instruction of people working for the sick and wounded soldiers. But when 1905 saw the successful launching of a movement, under royal patronage, for a British Red Cross Society for the Empire, the work of the St. Andrew's Ambulance Association in Scotland was provided for. The Association very patriotically decided to help the Red Cross Society to found a Scottish branch, and being, therefore, unable to use the money previously handed over to it, has now agreed to make the above application. The Red Cross Branch in Scotland will look after Scottish interests with the transferred funds, and £3,500 has been retained to meet prospective expenses in relation to the training of the St. Andrew's Ambulance Corps. The previous management and present proposal show such good sense that the Association should be congratulated on achieving the rare combination of patriotism and sound finance.

### Prevention of Abortion in Belgium.

A Committee, consisting of M. Daels, pharmacist, Dr. Casse, vice-president of the Belgian Royal Society of State Medicine and Medical Topography, M. Rops, lawyer, and Dr. Dupureux, secretary, was recently appointed by the above-mentioned society to inquire into the measures that should be taken to prevent the diminution in the birth-rate brought about by illicit methods. On October 23 these gentlemen issued their report to a general meeting of the Society, and the following are the conclusions at which they arrive. The best way of dealing with the present situation is by a propaganda by voice and pen. Laws are useful, but only protect those who accept their effects. The law against alcoholism is but the reflection of the revolt of public opinion against this anti-social vice. That which combats it best is a propaganda and the concentration of activities financed by the public authorities. The ravages of alcoholism have diminished not so much as the result of the law as by the effects of the goodwill of some and the enthusiasm of others. Societies should, therefore, be formed, newspapers founded, and tracts distributed so that their good advice may penetrate everywhere. Doctors, moralists, priests, and teachers of all kinds must unite in the good work, and the question must be thrashed out unceasingly. Help should be given to necessitous mothers, whether married or not. Laws should be passed placing the natural child, from the social and juridical point of view, on a level more nearly approaching that of the legitimate child. The manufacture, the advertisement, the sale, as well as the use of anti-conceptional and abortificient apparatus should be prevented by law, and the punishment of offences against it should be deferred to the correctional tribunal. A reduction in taxation should be made to parents with numerous children, as well as a pension to the father at the age of sixty, the pension being made proportional to the number of children in the case of poor families. Succession duties should be greatly increased in cases where the inheritance does not pass directly from father to son. Moneys so obtained should be devoted solely towards defraying the cost of the scheme outlined above.

### An Unsatisfactory Appointment.

On November 1 the London County Council once more demonstrated how unfit a popularly elected assembly of party politicians is to deal with matters which require expert professional knowledge. The General Purposes Committee of the Council sent up the name of a well-known deputy coroner, Mr. Ingleby Oddie, for the coronership left vacant by the death of Mr. Danford Thomas. This Committee had interviewed the candidates, and had carefully considered the claims of Mr. Walter Schröder, who has for some years done Mr. Danford Thomas's work. Mr. Schröder, all admit, has discharged the duties extremely well; but he has several important disqualifications. He is fifty-five, where thirty-five to fifty were indicated as desirable limits in the Council's own advertisement of the post. He is neither a qualified medical man nor a lawyer, whereas Mr. Oddie is both, and is also within the prescribed limits of age. The plain fact is that Mr. Thomas was for years incapable of carrying out the duties of his well-salaried office, but refused to resign. He appointed as his deputy Mr. Schröder, whose qualifications, other than his own common-sense, were *nil*; and the County Council now deliberately sanctions this monstrous precedent by over-ruling the recommendation of its own Committee. Probably Mr. Schröder will continue to discharge his duties with the same zeal and thoroughness which he has displayed hitherto. But the real point is not the personal one; it is that the Council by its action openly discourages medical men and barristers, and those who are both, from qualifying themselves for the important and responsible duties of a coroner. To place these quasi-judicial offices practically within the gift of those who already occupy them is deliberately to invite the lax standard of duty which, unfortunately, Mr. Danford Thomas was permitted to adopt.

